# Dietary intake and plasma concentrations of PUFAs in childhood and adolescence in relation to asthma and lung function up to adulthood

**DOI:** 10.1093/ajcn/nqab427

**Published:** 2021-12-29

**Authors:** Sandra Ekström, Emmanouela Sdona, Susanna Klevebro, Jenny Hallberg, Antonios Georgelis, Inger Kull, Erik Melén, Ulf Risérus, Anna Bergström

**Affiliations:** Center for Occupational and Environmental Medicine, Region Stockholm, Stockholm, Sweden; Institute of Environmental Medicine, Karolinska Institute, Stockholm, Sweden; Institute of Environmental Medicine, Karolinska Institute, Stockholm, Sweden; Department of Clinical Science and Education, Södersjukhuset, Karolinska Institute, Stockholm, Sweden; Sachs’ Children and Youth Hospital, Södersjukhuset, Stockholm, Sweden; Institute of Environmental Medicine, Karolinska Institute, Stockholm, Sweden; Sachs’ Children and Youth Hospital, Södersjukhuset, Stockholm, Sweden; Center for Occupational and Environmental Medicine, Region Stockholm, Stockholm, Sweden; Institute of Environmental Medicine, Karolinska Institute, Stockholm, Sweden; Department of Clinical Science and Education, Södersjukhuset, Karolinska Institute, Stockholm, Sweden; Sachs’ Children and Youth Hospital, Södersjukhuset, Stockholm, Sweden; Institute of Environmental Medicine, Karolinska Institute, Stockholm, Sweden; Department of Clinical Science and Education, Södersjukhuset, Karolinska Institute, Stockholm, Sweden; Sachs’ Children and Youth Hospital, Södersjukhuset, Stockholm, Sweden; Clinical Nutrition and Metabolism, Department of Public Health and Caring Sciences, Uppsala University, Uppsala, Sweden; Center for Occupational and Environmental Medicine, Region Stockholm, Stockholm, Sweden; Institute of Environmental Medicine, Karolinska Institute, Stockholm, Sweden

**Keywords:** asthma, childhood, dietary intake, longitudinal cohort, lung function, plasma proportions, polyunsaturated fatty acids, young adulthood

## Abstract

**Background:**

PUFAs may influence the risk of asthma; however, long-term prospective studies including objective biomarkers of PUFA intake are lacking.

**Objectives:**

The objective was to investigate the role of dietary intake and plasma concentrations of n–3 and n–6 (ω-3 and ω-6) PUFAs in childhood and adolescence for the development of asthma and lung function up to young adulthood.

**Methods:**

The study included participants from the Swedish prospective birth cohort BAMSE. Dietary intake of PUFAs was calculated from FFQs (*n* = 1992) and plasma proportions of PUFAs were measured in phospholipids (*n* = 831). We analyzed the n–3 PUFA α-linolenic acid (ALA; 18:3n–3); the sum of very-long-chain (VLC) n–3 PUFAs: EPA (20:5n–3), DHA (22:6n–3), and docosapentaenoic acid (22:5n–3); and the n–6 PUFAs linoleic acid (LA; 18:2n–6) and arachidonic acid (AA; 20:4n–6). Asthma was assessed by questionnaires at 8, 16, and 24 y and lung function was measured by spirometry at 24 y.

**Results:**

A high (≥median) self-reported dietary intake of LA at 8 y and AA at 16 y was associated with increased risk of prevalent asthma at 24 y (OR: 1.41; 95% CI: 1.10, 1.82 and OR: 1.32; 95% CI: 1.02, 1.70, respectively). In contrast, plasma proportions of ALA, ∑VLC n–3 PUFAs, and AA at 8 y, as well as LA at 16 y, were inversely associated with prevalent asthma at 24 y (e.g., OR: 0.55; 95% CI: 0.38, 0.81 for ∑VLC n–3 PUFAs). No consistent associations were observed with lung function.

**Conclusions:**

High dietary intake of certain n–6 PUFAs in childhood or adolescence may be associated with increased risk of asthma up to young adulthood, whereas dietary biomarkers of certain n–3 and n–6 PUFAs in plasma may be associated with decreased risk. Thus, the role of diet compared with altered metabolism of PUFAs needs further investigation to improve dietary preventive strategies for asthma.

## Introduction

The prevalence of allergic diseases including asthma has increased rapidly since the 1950s, with children and young adults bearing the greatest burden of this trend (1). Results from observational and experimental studies have suggested that very-long-chain (VLC) omega-3 (n–3) PUFAs, present in oily fish, may decrease the risk of asthma and other allergic diseases owing to their anti-inflammatory and immunomodulatory properties (2, 3). In contrast, omega-6 (n–6) PUFAs have been suggested to increase the risk of allergic disease (2), with some cross-sectional studies linking dietary intake or plasma concentrations of linoleic acid (LA; 18:2n–6) (present in, e.g., vegetable oils and nuts) or total n–6 PUFAs to increased risk of allergic disease (4–6). However, the mechanisms of n–6 fatty acids have been shown to be complex, and several studies have also found inverse associations between n–6 fatty acids and allergic disease (7–11). Thus, the role of n–6 PUFAs clearly needs further investigation in relation to allergic disease.

Whereas many studies on PUFAs and allergic disease have focused on maternal supplementation or dietary intake of fish and PUFAs in pregnancy (12–15), fewer studies have investigated the role of intakes during childhood. Longitudinal analyses from the Barn, Allergi, Miljö, Stockholm, Epidemiologi (BAMSE) birth cohort have previously shown that regular intake of fish in infancy, as well as biomarkers of fish intake at school age, can reduce the risk of allergic disease including asthma ≤12 and ≤16 y of age, respectively (11, 16). In addition, a recent study based on the UK Avon Longitudinal Study of Parents and Children (ALSPAC) birth cohort, with replication in the BAMSE birth cohort, found that this association may be modified by fatty acid desaturase (*FADS*) genotype (17). The results showed that higher intake of the VLC n–3 PUFAs EPA (20:5n–3) and DHA (22:6n–3) from fish in childhood was inversely associated with incident asthma up to adolescence only among participants with a common genetic variant (minor G allele), which has previously been associated with lower plasma concentrations of VLC n–3 PUFAs (17).

Taken together, findings from the BAMSE birth cohort and other previous studies have indicated that long-chain PUFAs may influence subsequent development of asthma in childhood, suggesting a possible favorable role of VLC n–3 PUFAs. However, it remains unknown if this influence persists into adulthood, and the role of the major n–6 PUFAs in plasma, LA and arachidonic acid (AA; 20:4n–6), respectively, is yet unclear. In addition, it is still unclear if long-chain PUFAs found in fish and vegetable oils are associated with lung function or other objective markers of disease severity. Therefore, the aim of the present study was to investigate the role of dietary intake and plasma concentrations of long-chain n–3 PUFAs and the 2 major n–6 PUFAs (LA and AA) in childhood and adolescence for the development of asthma and lung function up to young adulthood.

## Methods

### Study population and study design

The study population includes participants from the prospective birth cohort BAMSE, which has been described in detail previously (18). The BAMSE study includes 4089 participants, born between 1994 and 1996 in the northwestern and central parts of Stockholm, Sweden. The children were subsequently followed with repeated questionnaires focusing on lifestyle, environmental exposures, and allergic diseases ≤24 y of age. The response rate has remained persistently high throughout the follow-ups, with 3064 (75%) of the baseline participants answering the 24-y questionnaire.

Repeated clinical examinations including blood sampling, lung function testing, and anthropometric assessments have been performed at 4, 8, 16, and 24 y of age in a majority of the participants [*n* = 2271 (56%) at 24 y] (19). Blood samples were analyzed for IgE antibodies with ImmunoCAP™ (Thermo Fisher Scientific). In a subsample of 940 and 939 participants at 8 and 16 y, respectively, blood samples were in addition analyzed for fatty acids in plasma phospholipids (as will be described). Lung function was measured by spirometry at 8, 16, and 24 y (see the **Supplemental Methods**) (20). The results were analyzed as age-, height-, and gender-adjusted values of forced expiratory volume in 1 s (FEV_1_) and forced vital capacity (FVC) (in mL), FEV_1_/FVC (%), and by *z* scores using the Global Lung Initiative reference values (21). The study was approved by the Swedish Ethical Review Authority (approval number 2016/1380-31/2). Participants provided written informed consent.

### Exposure assessment

Diet was assessed by FFQs at 8 y and 16 y. At 8 y, the FFQ covered consumption of 98 foods and was filled out by the parents (57%) or the parents together with the child (40%). At 16 y, the adolescents themselves answered a web-based FFQ (TeenMeal-Q) including 107 food items. Similar FFQs to the ones used at 8 and 16 y have been validated in adults with correlation coefficients for total energy-adjusted PUFAs of 0.49 and 0.35, respectively (22, 23). Dietary intake of PUFAs was calculated using nutrient content per serving obtained from the Swedish Food Agency (24) multiplied by reported frequency of consumption. Intakes of PUFAs were adjusted by energy intake using the residual method and presented per mean energy intake (1900 kcal) (25).

Fatty acids in plasma phospholipids were analyzed at 8 y (*n* = 940) and 16 y (*n* = 939) using GC as previously described (11). Concentrations were expressed in relative amounts as proportions of total fatty acids comprising the 15 different fatty acids obtained (see the Supplemental Methods). In the present study, we analyzed the n–3 fatty acid α-linolenic acid (ALA; 18:3n–3); the VLC n–3 fatty acids EPA, DHA, and docosapentaenoic acid (DPA; 22:5n–3); and the major n–6 fatty acids LA and AA. The VLC n–3 fatty acids were analyzed together owing to previously observed high intercorrelation and shared dietary sources (11). For dietary intakes of PUFAs, the n–6:n–3 ratio was in addition calculated by dividing the major n–6 PUFAs LA and AA by the sum of the n–3 PUFAs (ALA, EPA, DPA, and DHA).

### Definition of outcomes

Asthma was assessed at 8, 16, and 24 y based on questionnaires (parental reports at 8 y and self-reports at 16 and 24 y). Asthma was defined as fulfilling ≥2 out of 3 criteria: doctor's diagnosed asthma (ever up to the date of the specific questionnaire), ≥1 episode of wheeze and/or breathing difficulties in the last 12 mo, and/or any asthma medication use in the last 12 mo (26). Incident asthma between 8 and 24 y was defined as fulfilling the definition of asthma at 24 y, but not at 8 y.

### Assessment of covariates

Information on covariates was obtained from the baseline questionnaire (sex, living area at birth, allergic heredity, parental occupation, maternal smoking in pregnancy and/or infancy), the 1-y questionnaire (breastfeeding, fish intake in infancy), the 8-y questionnaire (parental birth country, fish intake, antioxidant intake, vitamin D intake, dietary supplements, overweight), the 16-y questionnaire (fish intake, n–3 supplements, smoking, overweight, physical activity), the 24-y questionnaire (smoking, overweight, physical activity), and the Swedish Medical Birth Register (maternal BMI in early pregnancy).

Allergic sensitization at 24 y was defined as a positive Phadiatop [cat, dog, horse, timothy, birch, mugwort, *Dermatophagioides pternyssinus* (house dust mite) and *Cladosporium* (mold)] or Fx5 (cow milk, egg, cod, wheat, peanut, soy) result (IgE ≥0.35 kU/L). The *FADS* single nucleotide polymorphism (rs1535 A/G) was available from genome-wide association study (GWAS) data in BAMSE for a total of 2712 subjects (27). The Supplemental Methods present full details of the *FADS* analysis and definitions of the covariates.

### Statistical analyses

Correlations between individual plasma PUFAs at 16 y were analyzed by Spearman rank correlation with Bonferroni multiple test correction. Spearman rank correlation was also used to analyze correlations between energy-adjusted dietary intakes of PUFAs and plasma proportions of PUFAs at 16 y. Correlations between plasma fatty acids as well as between plasma and dietary fatty acids at 8 y have been previously reported in the BAMSE study (11).

Dietary and plasma fatty acids were analyzed separately at 8 and 16 y and categorized into 2 groups (referred to as low and high levels) based on the median value for each PUFA. Differences in selected baseline and lifestyle characteristics were analyzed in relation to mean plasma proportions of ∑VLC n–3 PUFAs at 8 and 16 y by chi-square test. Associations between dietary and plasma PUFAs and asthma prevalence and incidence were analyzed via logistic regression, whereas lung function was analyzed using linear regression, using the lower median of the exposures as the reference. To fully explore the associations between plasma proportions of PUFAs and asthma, plasma proportions of each PUFA were further analyzed as continuous variables flexibly modeled using restricted cubic splines with 3 knots.

In order to utilize the longitudinal design with repeated exposure assessment, associations between plasma proportions of PUFAs and prevalent asthma were further analyzed longitudinally using generalized estimating equations with a binomial family, a logit link function, and an unstructured correlation matrix. In these models, PUFA was handled as an updated lagged exposure, i.e., PUFA concentrations at 8 y were modeled against asthma at 8 and 16 y and PUFA concentrations at 16 y were modeled against asthma at 24 y. Interaction terms between each PUFA and the time indicator variable were used to estimate age-specific associations at 8, 16, and 24 y.

As a sensitivity analysis, associations were further adjusted for early symptoms of allergic disease (wheeze and eczema ≤2 y of age, assessed in the 2-y follow-up) in order to investigate potential disease-related modification of exposure. In addition, participants with reported reactions to fish at 8 and 16 y or avoidance of fish due to previous reactions were excluded to avoid confounding by indication (i.e., fish-allergic patients having lower PUFA concentrations due to avoidance).

Analyses were adjusted for previously established risk factors for asthma: sex, allergic heredity, socioeconomic status (assessed by parental occupation at baseline), and maternal smoking in pregnancy and/or early infancy. We further evaluated additional potential confounders (see Assessment of covariates) by including them one by one in the crude regression model between ∑VLC n–3 PUFAs at 8 and 16 y and prevalent asthma at 24 y. None of these variables affected the estimate >5% and they were therefore not included in the final models. The analyses of lung function (mL) were in addition adjusted for age and height. To investigate potential effect modification by gender, allergic sensitization, or *FADS* genotype, stratified analyses were performed.

Participants were included in the analyses if information on dietary intake or plasma concentrations of fatty acids at 8 and 16 y, and asthma or lung function at 24 y were available. For dietary PUFAs, there were 1992 participants in the analyses with asthma (1957 in the fully adjusted model) and 1428 participants in the analyses with lung function (1399 in the fully adjusted model). For plasma PUFAs, there were 831 participants in the analyses with asthma (825 in the fully adjusted models) and 639 participants in the analyses with lung function (632 in the fully adjusted models). **Supplemental Figure 1** shows a flowchart of the included participants.

## Results

### Dietary intake and plasma proportions of PUFAs

The study populations with information on dietary intakes of PUFAs (*n* = 1992) and plasma proportions of PUFAs (*n* = 831) were generally comparable with the original BAMSE cohort, although with a somewhat lower proportion of males in the study population with measured plasma proportions of PUFAs (44.0% compared with 50.5%) (**Supplemental Table 1**).

Median dietary intakes of ∑VLC n–3 PUFAs were 0.24 g/1900 kcal and 0.27 g/1900 kcal at 8 and 16 y, respectively, and slightly higher in girls than in boys at 8 y (*P* = 0.02) (**Supplemental Table 2**). There was no difference in intake of ∑VLC n–3 PUFAs in relation to *FADS* genotype. At 16 y, energy-adjusted intake of LA and n–6:n–3 ratio were higher among females than among males (*P* < 0.001).

Median plasma proportions of all PUFAs increased from 8 to 16 y. ∑VLC n–3 PUFAs increased from 3.3% to 5.4%, LA from 21.2% to 21.9%, and AA from 5.6 to 8.9% (all *P* < 0.001). There were no gender differences in the proportions of ∑VLC n–3 PUFAs, whereas AA was higher in males than in females at both ages (*P* = 0.02 and *P* < 0.001, respectively) (Supplemental Table 2).

**Supplemental Table 3** presents correlation coefficients between plasma proportions of PUFAs at 16 y. ∑VLC n–3 PUFAs was weakly negatively correlated with LA (*r* = −0.35, *P* < 0.001) and weakly positively correlated with AA (*r* = 0.29, *P* < 0.001) but not with ALA. The correlations between reported dietary intakes and plasma proportions of PUFAs at 16 y varied from −0.04 for ALA to 0.31 for DHA and ∑VLC n–3 PUFAs (**Supplemental Table 4**).

A high (above median) plasma proportion of combined ∑VLC n–3 PUFAs at 8 and 16 y was associated with higher socioeconomic status (e.g., in terms of living area at birth and parental work), *FADS* genotype, fish intake, and lower proportion of overweight (all *P <* 0.05) (**Table 1**). **Supplemental Table 5** presents plasma proportions of ∑VLC n–3 PUFAs at 8 and 16 y in relation to these factors. The median plasma proportion of ∑VLC n–3 PUFAs was 4.58% among individuals with *FADS* genotype AA, compared with 4.30% among individuals with the GA/GG genotype (*P* < 0.001).

**TABLE 1:** Description of baseline and lifestyle characteristics in relation to median plasma proportions of VLC n–3 PUFAs at 8 and 16 y in the study population^1^

Variables	Low^2^ ∑VLC n–3 PUFAs (<4.41%) (*n* = 416)	High^2^ ∑VLC n–3 PUFAs (≥4.41%) (*n* = 415)	*P* value^3^
Male sex (*n* = 831)	190 (45.7)	176 (42.4)	0.34
Living area at birth (*n* = 827)			
Urban^4^	105 (25.4)	165 (39.9)	
Suburban^4^	308 (74.6)	249 (60.1)	<0.001
Parental professional worker at baseline (*n* = 826)	349 (84.7)	379 (91.6)	0.002
Maternal overweight in early pregnancy (*n* = 811)	83 (20.5)	85 (20.9)	0.88
Maternal smoking in pregnancy and/or infancy (*n* = 830)	56 (13.5)	38 (9.2)	0.049
Parental origin outside Scandinavia (*n* = 831)	69 (16.6)	64 (15.4)	0.65
*FADS* genotype (*n* = 762), GA/GG (rs1535)	268 (70.0)	214 (56.5)	<0.001
Breastfeeding (*n* = 816), ≥4 mo	324 (79.6)	332 (81.2)	0.57
Fish intake at 1 y (*n* = 816), ≥2 times/mo	327 (80.3)	332 (81.2)	0.76
Fish intake at 8 y (*n* = 831), ≥2 times/wk	146 (35.1)	187 (45.1)	0.003
Fish intake at 16 y (*n* = 830), ≥1–2 times/wk	252 (60.6)	327 (79.0)	<0.001
ω-3 supplements at 16 y^5^ (*n* = 718)	18 (5.1)	25 (6.9)	0.31
Smoking at 16 y^6^ (*n* = 831)	48 (11.5)	40 (9.6)	0.37
Smoking at 24 y^6^ (*n* = 830)	97 (23.3)	77 (18.6)	0.10
Overweight at 8 y (*n* = 831)	75 (18.0)	95 (22.9)	0.08
Overweight at 16 y (*n* = 829)	66 (15.9)	69 (16.7)	0.77
Overweight at 24 y (*n* = 686)	93 (27.1)	71 (20.7)	0.049
High physical activity at 16 y^7^ (*n* = 808)	284 (69.8)	295 (73.6)	0.23
High physical activity at 24 y^7^ (*n* = 680)	192 (55.5)	193 (57.8)	0.55

1 Study population, *n* = 831. Values are *n* (%) unless indicated otherwise. Differences between groups with high and low levels were tested by the chi-square test. *FADS*, fatty acid desaturase; ∑VLC n–3 PUFAs, sum of very-long-chain n–3 PUFAs [EPA (20:5n–3), docosapentaenoic acid (22:5n–3), and DHA (22:6n–3)].

2 Low and high categories were defined by the median value (4.41%).

3 *P* value obtained by the chi-square test.

4 Urban: central parts of Stockholm (Norrmalm); suburban: northwestern parts of Stockholm county (the municipalities Järfälla, Solna, and Sundbyberg).

5 Everyday users.

6 Daily or occasional smoking.

7 Levels of physical activity according to International Physical Activity Questionnaire guidelines at the time of the questionnaire at 24 y. High: ≥7 h/wk of moderate to vigorous activity or ≥3.5 h/wk of vigorous activity.

### Associations of dietary intakes and plasma proportions of PUFAs with prevalence and incidence of asthma from 8 to 24 y

Among the 1992 participants with information on dietary PUFAs and asthma, 291 (14.6%) fulfilled the definition of asthma at 24 y (16.0% in females and 13.1% in males, *P* = 0.07), with 169 of 1770 (9.6%) incident cases between 8 and 24 y. The prevalence of asthma was somewhat higher in the study population with plasma PUFA data available (**Supplemental Table 6**).

In analyses of dietary PUFAs, high (≥median) intake of the n–6 PUFA LA at 8 y, but not 16 y, was associated with increased risk of prevalent asthma at 24 y (OR: 1.41; 95% CI: 1.10, 1.82). In contrast, intakes of AA at 16 y, but not 8 y, were associated with increased risk of prevalent asthma at 24 y (OR: 1.32; 95% CI: 1.02, 1.70). A high n–6:n–3 ratio at 8 y was further associated with incident asthma from 8 to 24 y, whereas no association was observed at 16 y (**Table 2**). No association was observed between dietary n–3 PUFAs and asthma.

**TABLE 2:** Associations of dietary intakes and plasma proportions of PUFAs at 8 and 16 y with prevalence and incidence of asthma at 24 y analyzed by logistic regression^1^

	Prevalent asthma 24 y	Incident asthma 8–24 y
Dietary PUFAs (mg/1900 kcal), ≥median vs. <median (*n* = 1992)^2^		
n–3 PUFAs		
ALA		
8 y (≥1145.0)	1.03 (0.80, 1.32)	0.89 (0.65, 1.24)
16 y (≥1330.7)	0.95 (0.74, 1.23)	1.03 (0.75, 1.42)
∑VLC n–3		
8 y (≥234.5)	1.01 (0.78, 1.30)	0.83 (0.60, 1.15)
16 y (≥268.8)	0.97 (0.75, 1.25)	0.94 (0.68, 1.30)
n–6 PUFAs		
LA		
8 y (≥6249.1)	1.41 (1.10, 1.82)	1.32 (0.96, 1.83)
16 y (≥7850.3)	0.92 (0.71, 1.18)	0.94 (0.68, 1.30)
AA		
8 y (≥70.7)	0.94 (0.73, 1.21)	0.95 (0.69, 1.31)
16 y (≥77.0)	1.32 (1.02, 1.70)	1.19 (0.86, 1.64)
n–6:n–3 ratio		
8 y (≥4.6)	1.15 (0.89, 1.48)	1.46 (1.05, 2.03)
16 y (≥4.8)	0.96 (0.75, 1.24)	0.98 (0.71, 1.36)
Plasma PUFAs (% of total) , ≥median vs. <median (*n* = 831)^3^		
n–3 PUFAs		
ALA		
8 y (≥0.24)	0.63 (0.43, 0.91)	0.63 (0.39, 1.04)
16 y (≥0.28)	0.82 (0.56, 1.18)	0.71 (0.44, 1.16)
∑VLC n–3		
8 y (≥3.3)	0.55 (0.38, 0.81)	0.59 (0.36, 0.97)
16 y (≥5.4)	0.74 (0.51, 1.08)	0.59 (0.36, 0.98)
n–6 PUFAs		
LA		
8 y (≥21.2)	0.76 (0.52, 1.10)	0.76 (0.47, 1.23)
16 y (≥21.9)	0.68 (0.47, 0.99)	0.84 (0.52, 1.36)
AA		
8 y (≥5.6)	0.65 (0.44, 0.94)	0.69 (0.42, 1.13)
16 y (≥8.9)	1.04 (0.72, 1.52)	1.07 (0.66, 1.75)

1 Values are OR (95% CI). ORs were estimated by logistic regression models adjusted for sex, allergic heredity, parental occupation at baseline, and maternal smoking in pregnancy and/or infancy. AA, arachidonic acid (20:4n–6); ALA, α-linolenic acid (18:3n–3); DPA, docosapentaenoic acid (22:5n–3); LA, linoleic acid (18:2n–6); n–6:n–3 ratio, sum of LA and AA divided by sum of ALA, EPA (20:5n–3), DPA, and DHA (22:6n–3); ∑VLC n–3, sum of very-long-chain n–3 PUFAs (EPA, DPA, and DHA).

2 Prevalent asthma 24 y, *n* = 291 of 1992; incident asthma 8–24 y, *n* = 169 of 1770.

3 Prevalent asthma 24 y, *n* = 142 of 831; incident asthma 8–24 y, *n* = 76 of 715.

Analyses of plasma proportion of PUFAs showed that a high (≥median) plasma proportion of n–3 PUFAs (ALA and ∑VLC n–3 PUFAs) at 8 y was inversely associated with prevalent asthma at 24 y (e.g., OR: 0.55; 95% CI: 0.38, 0.81 for ∑VLC n–3 PUFAs) (Table 2). The same trend was observed for ∑VLC n–3 PUFAs at 16 y although it was only significant for incident asthma. Further, high plasma proportions of the n–6 PUFAs AA at 8 y and LA at 16 y were associated with decreased risk of prevalent asthma at 24 y (OR: 0.68; 95% CI: 0.47, 0.99 and OR: 0.65; 95% CI: 0.44, 0.94, respectively).

**FIGURE 1:**
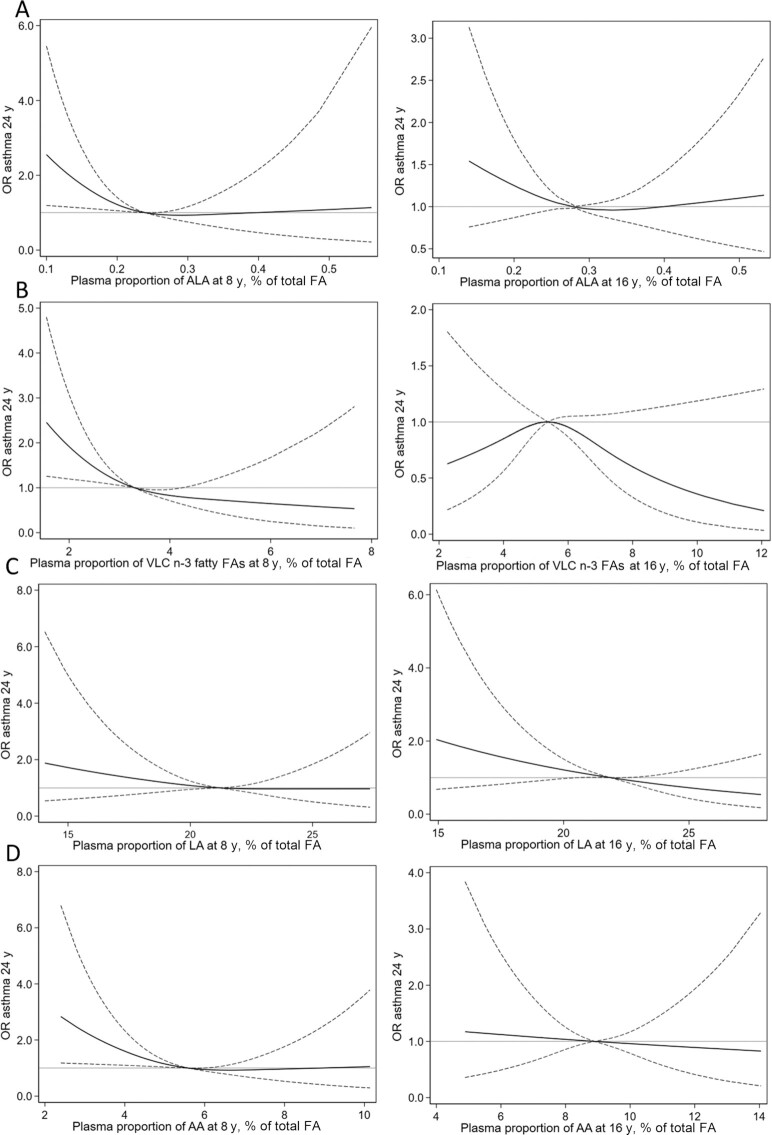
Associations between continuous plasma proportions of ALA (18:3n–3) (A), sum of VLC n–3 PUFAs [EPA (20:5n–3), docosapentaenoic acid (22:5n–3), and DHA (22:6n–3)] (B), LA (18:2n–6) (C), and AA (20:4n–6) (D) and prevalent asthma at 24 y, flexibly modeled using restricted cubic splines with 3 knots (*n* = 831). The left panel represents PUFA proportions at 8 y and the right panel represents PUFA proportions at 16 y. ORs and 95% CIs were estimated using logistic regression adjusted for sex, allergic heredity, parental occupation at baseline, and maternal smoking in pregnancy and/or infancy, with the median proportions for each PUFA as the reference. The solid lines represent ORs and the dashed lines represent 95% CIs. AA, arachidonic acid; ALA, α-linolenic acid; FA, fatty acid; LA, linoleic acid; VLC, very-long-chain.

**Figure 1** shows the flexibly modeled associations between plasma proportions of PUFAs at 8 and 16 y and prevalent asthma at 24 y. Evidence for a nonlinear association was observed for all PUFAs except LA at 8 y (all *P* < 0.05), but not at 16 y. Compared with median values, proportions of ALA, ∑VLC n–3 PUFAs, and AA below the median at 8 y were progressively associated with increased risk of asthma at 24 y, whereas no significant associations were observed for proportions above the median.

**FIGURE 2:**
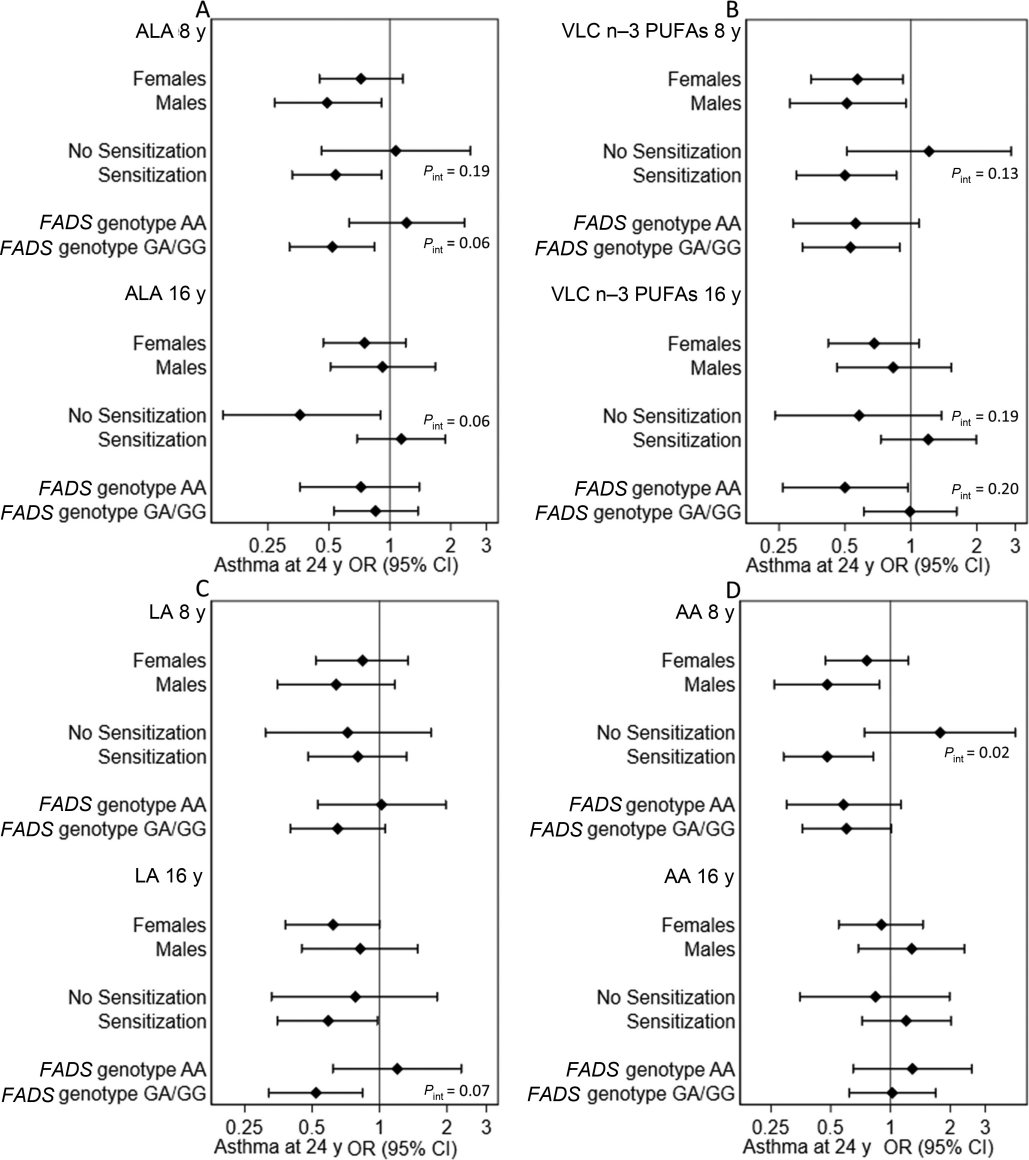
Associations between high plasma proportions (≥median) of ALA (18:3n–3) (A), sum of VLC n–3 PUFAs [EPA (20:5n–3), docosapentaenoic acid (22:5n–3), and DHA (22:6n–3)] (B), LA (18:2n–6) (C), and AA (20:4n–6) (D) at 8 and 16 y and prevalent asthma at 24 y, stratified by gender (*n* = 831), allergic sensitization (*n* = 685), and *FADS* genotype (*n* = 762). Analyses are adjusted for sex, allergic heredity, parental occupation at baseline, and maternal smoking in pregnancy and/or infancy. *P* values for interactions ≤0.2 are shown in the figure. All other *P* values for interaction were >0.2. AA, arachidonic acid; ALA, α-linolenic acid; *FADS*, fatty acid desaturase; LA, linoleic acid; VLC, very-long-chain.

Stratified analyses by sex, allergic sensitization, and *FADS* genotype showed that there was a significant interaction between dietary intake of ALA at 8 y and allergic sensitization (*P* = 0.005); high intake of ALA was associated with reduced risk of prevalent asthma at 24 y among participants without allergic sensitization (OR: 0.43; 95% CI: 0.23, 0.79), but not among participants with allergic sensitization (OR: 1.23; 95% CI: 0.87, 1.73). For dietary PUFAs at 16 y, there was a significant interaction between AA and allergic sensitization (*P* = 0.01), where an increased risk of asthma was observed only among sensitized individuals (OR: 1.72; 95% CI: 1.21, 2.45 compared with OR: 0.75; 95% CI: 0.42, 1.33 among nonsensitized). No other significant differences were observed in the association between dietary PUFAs and asthma in relation to these factors (data not shown). For plasma proportions of PUFAs (**Figure 2**), there was a significant interaction between AA at 8 y and allergic sensitization (*P* = 0.02), with an inverse association with asthma only among sensitized individuals. No other significant differences were observed in relation to these factors.

### Associations between plasma proportions of PUFAs and asthma from childhood to young adulthood using an updated lagged exposure model

Results of the updated lagged exposure model of plasma proportions of PUFAs in the longitudinal analysis of asthma from 8 to 24 y showed that a high plasma proportion of ∑VLC n–3 PUFAs was associated with reduced risk of asthma ≤24 y (overall OR: 0.70; 95% CI: 0.57, 0.87). The association was similar at 8, 16, and 24 y (*P* values for interactions with time = 0.90 for 16 y compared with 8 y and 0.69 for 24 y compared with 8 y). There were no statistically significant associations between ALA, LA, or AA and asthma ≤24 y in this model (**Table 3**).

**TABLE 3:** Longitudinal associations between plasma proportions of PUFAs and asthma from childhood (8 y) to young adulthood (24 y) analyzed by generalized estimating equation models^1^

Plasma PUFAs (% of total), ≥median vs. <median	Asthma 8 y	Asthma 16 y		Asthma 24 y		Overall
	OR^2^ (95% CI)	OR^2^ (95% CI)	*P*_int_^3^	OR^2^ (95% CI)	*P*_int_^4^	OR^2^ (95% CI)
n–3 PUFAs						
ALA	1.09 (0.75, 1.57)	1.14 (0.85, 1.52)	0.83	0.86 (0.64, 1.15)	0.32	1.00 (0.81, 1.22)
∑VLC n–3	0.74 (0.51, 1.08)	0.72 (0.54, 0.97)	0.90	0.68 (0.50, 0.91)	0.69	0.70 (0.57, 0.87)
n–6 PUFAs						
LA	0.97 (0.67, 1.40)	1.13 (0.85, 1.52)	0.41	0.80 (0.60, 1.08)	0.43	0.95 (0.77, 1.16)
AA	0.98 (0.68, 1.42)	0.85 (0.63, 1.15)	0.48	0.93 (0.69, 1.25)	0.82	0.91 (0.73, 1.13)

1 *n* = 831. Adjusted ORs and 95% CIs are estimated using generalized estimating equations with an updated lagged exposure (PUFAs at 8 y are modeled against asthma at 8 and 16 y, PUFAs at 16 y are modeled against asthma at 24 y). AA, arachidonic acid (20:4n–6); ALA, α-linolenic acid (18:3n–3); LA, linoleic acid (18:2n–6); ∑VLC n–3, sum of very-long-chain n–3 PUFAs [EPA (20:5n–3), docosapentaenoic acid (22:5n–3), and DHA (22:6n–3)].

2 Adjusted for sex, allergic heredity, parental occupation at baseline, and maternal smoking in pregnancy and/or infancy.

3 *P* value for interaction 16 y compared with 8 y.

4 *P* value for interaction 24 y compared with 8 y.

### Associations of dietary intake and plasma proportions of PUFAs at 8 and 16 y with lung function at 24 y

Relative lung function values (*z* scores) were higher in females than in males [e.g., mean FEV_1_-*z*: −0.16 (3506 mL) compared with −0.34 (4743 mL) at 24 y, *P* = 0.001] (Supplemental Table 6). There was no significant association between dietary or plasma PUFAs at 8 or 16 y and lung function at 24 y, except for an association between plasma ∑VLC n–3 PUFAs at 8 y and higher FVC (86.0 mL; 95% CI: 6.9, 165.1 mL) but lower FEV_1_:FVC ratio (−1.35 %; 95% CI: −2.27, −0.43 %) (**Table 4**). Analyses using *z* scores instead of milliliters gave similar results (data not shown).

**TABLE 4:** Association of dietary and plasma PUFAs at 8 and 16 y with lung function at 24 y analyzed with linear regression models^1^

	FEV_1_, mL	FVC, mL	FEV_1_/FVC, %
Dietary PUFAs (mg/1900 kcal) (*n* = 1428), ≥median vs. <median			
n–3 PUFAs			
ALA			
8 y (≥1145.0)	−25.6 (−70.3, 19.1)	−52.3 (−106.4, 1.74)	0.34 (−0.28, 0.96)
16 y (≥1330.7)	−31.2 (−75.9, 13.6)	−49.2 (−103.3, 4.90)	0.10 (−0.53, 0.72)
∑VLC n–3			
8 y (≥234.5)	5.09 (−40.1, 50.2)	16.0 (−38.6, 70.7)	−0.25 (−0.88, 0.38)
16 y (≥268.8)	1.35 (−43.5, 46.2)	18.3 (−36.0, 72.5)	−0.34 (−0.96, 0.29)
n–6 PUFAs			
LA			
8 y (≥6249.1)	−0.03 (−44.9, 44.8)	−28.5 (−82.8, 25.7)	0.52 (−0.11, 1.14)
16 y (≥7850.3)	−6.52 (−51.6, 38.5)	−20.3 (−74.8, 34.2)	0.08 (−0.55, 0.71)
AA			
8 y (≥70.7)	−8.90 (−53.8, 36.0)	−0.68 (−55.0, 53.7)	−0.16 (−0.79, 0.46)
16 y (≥77.0)	0.83 (−44.3, 45.9)	8.66 (−45.9, 63.2)	−0.06 (−0.69, 0.57)
n–6:n–3 ratio			
8 y (≥4.6)	2.56 (−42.5, 47.6)	21.8 (−32.7, 76.2)	−0.20 (−0.83, 0.43)
16 y (≥4.8)	0.11 (−44.9, 45.1)	−5.23 (−59.6, 49.2)	0.12 (−0.51, 0.75)
Plasma PUFAs (% of total) (*n* = 639), ≥median vs. <median			
n–3 PUFAs			
ALA			
8 y (≥0.24)	−56.6 (−121.5, 8.2)	−52.0 (−131.4, 27.5)	−0.27 (−1.19, 0.66)
16 y (≥0.28)	10.3 (−54.7, 75.2)	−9.7 (−89.1, 69.8)	0.54 (−0.38, 1.47)
∑VLC n–3			
8 y (≥3.3)	−0.87 (−65.7, 64.0)	86.0 (6.9, 165.1)	−1.35 (−2.27, −0.43)
16 y (≥5.4)	−49.0 (−114.1, 16.0)	−11.7 (−91.5, 68.0)	−0.89 (−1.82, −0.04)
n–6 PUFAs			
LA			
8 y (≥21.2)	−50.5 (−115.3, 14.2)	−31.7 (−111.1, 47.7)	−0.35 (−1.27, 0.57)
16 y (≥21.9)	29.8 (−34.4, 94.1)	0.62 (−78.1, 79.4)	0.65 (−0.26, 1.57)
AA			
8 y (≥5.6)	24.8 (−40.8, 90.4)	55.7 (−24.6, 135.9)	−0.37 (−1.31, 0.56)
16 y (≥8.9)	−3.15 (−69.6, 63.3)	−4.3 (−85.6, 77.0)	−0.17 (−1.12, 0.77)

1 Values are β (95% CI). β-coefficients are estimated using linear regression models adjusted for height, age, sex, allergic heredity, parental occupation at baseline, and maternal smoking in pregnancy and/or infancy. AA, arachidonic acid (20:4n–6); ALA, α-linolenic acid (18:3n–3); DPA, docosapentaenoic acid (22:5n–3); FEV_1_, forced expiratory volume in 1 s; FVC, forced vital capacity; LA, linoleic acid (18:2n–6); n–6:n–3 ratio, sum of LA and AA divided by the sum of ALA, EPA (20:5n–3), DPA, and DHA (22:6n–3); ∑VLC n–3, sum of very-long-chain n–3 PUFAs (EPA, DPA, and DHA).

### Sensitivity analyses

The analyses of plasma proportion of PUFAs and prevalent asthma at 24 y (in Table 2) were in addition adjusted for early symptoms of allergic disease (wheeze and eczema ≤2 y of age) in order to investigate potential disease-related modification of exposure. The additional adjustment had no impact on the association between plasma proportion of ∑VLC n–3 PUFAs at 8 or 16 y and asthma at 24 y (OR: 0.58; 95% CI: 0.39, 0.85 for ∑VLC n–3 PUFAs at 8 y and OR: 0.68; 95% CI: 0.46, 1.01 for PUFAs at 16 y). In addition, excluding subjects who reported that they had an allergic reaction to fish or that they avoided fish because of allergy (*n* = 4 at 8 y and *n* = 9 at 16 y) had no impact on this association.

## Discussion

### Summary of results

In the present longitudinal cohort study, we observed that high self-reported dietary intakes of the n–6 fatty acids LA at 8 y and AA at 16 y were associated with increased risk of asthma at 24 y. In contrast, high plasma concentrations of these fatty acids (AA at 8 y and LA at 16 y) were associated with decreased risk. There was no association between self-reported dietary intake of n–3 PUFAs and asthma, whereas plasma concentrations of ALA and VLC n–3 PUFAs at 8 y were associated with decreased risk of asthma at 24 y. Overall, no consistent associations were observed between PUFAs and lung function.

### Comparison with previous studies

#### Dietary PUFAs and asthma

The current study is the first that we know of to analyze the association between repeated measures of dietary intakes, as well as plasma proportions, of PUFAs and asthma longitudinally from childhood up to young adulthood. Previous studies on dietary intake of n–3 and n–6 PUFAs in childhood in relation to asthma or asthma symptoms have observed inconsistent results, with both decreased risks (10), increased risks (28, 29), as well as no associations (30, 31) for both n–3 and n–6 PUFAs. These studies found no clear difference between n–3 and n–6 PUFAs in relation to these outcomes, whereas others have found that the n–6:n–3 ratio or intake of dietary products rich in n–6 PUFAs (e.g., margarine) may increase the risk of asthma (4, 32). The increased risk of asthma associated with higher dietary intake of n–6 PUFAs observed in our study was not confirmed when analyzing plasma concentrations of PUFAs. However, plasma concentrations of fatty acids reflect not only dietary intakes, but also endogenous metabolism (33), which may partly explain the observed differences. Overall, dietary and plasma VLC n–3 PUFAs are more strongly correlated than n–6 PUFAs, which was also seen in this study; however, this varies and depends on the lipid compartment used, dietary intake method, and population (34). For example, LA is an essential fatty acid that must be derived from diet, and is thus regarded as a valid and reliable biomarker of LA intake as assessed by dose-response studies and various cohort studies (35). ALA is also an essential fatty acid, but a weaker dietary biomarker than LA, possibly because ALA is more readily oxidized (36, 37). EPA and DHA are not essential, but the plasma concentrations are very good biomarkers of VLC n–3 PUFA intake from seafood. In addition, self-reported fatty acid intake using FFQ has several shortcomings.

In the present study, there were no consistent differences in the observed association related to gender, allergic sensitization, or *FADS* genotype. One recent longitudinal study on dietary VLC n–3 PUFAs from fish intake however observed a significant inverse association with incident asthma up to adolescence only among the minor allele G carriers of the *FADS* gene, associated with lower plasma concentrations of VLC n–3 PUFAs (17). Similar results were observed in a randomized trial on fish oil supplementation in pregnancy, where the strongest effect of supplementation in relation to childhood asthma was observed in children of mothers carrying the same *FADS* gene variant (12). It is therefore possible that any potential effect of VLC n–3 PUFAs is stronger among individuals with lower concentrations, although this could not be confirmed in the present study.

#### Plasma concentrations of PUFAs and asthma

Blood concentrations of VLC n–3 PUFAs in childhood have, in a few previous cross-sectional (10, 38), case-control (39), and longitudinal (40) analyses, been linked to a reduced risk of asthma or asthma symptoms up to early childhood, whereas some studies have found no association (5, 41, 42).

Regarding n–6 fatty acids, a Mendelian randomization study based on the UK Biobank concluded that genetically predicted LA concentrations might protect against asthma (43). However, results from traditional observational analyses have been conflicting (5, 9, 11, 44). In the BAMSE study, we have previously analyzed the association between plasma concentrations of PUFAs at 8 y and asthma ≤16 y (11), observing inverse associations of VLC n–3 PUFAs and AA with asthma. The results of the present study are in line with these findings and contribute unique longitudinal evidence regarding these associations up to adulthood.

#### PUFAs and lung function

Few previous studies have investigated the association between dietary or plasma PUFAs and lung function. In contrast to the present study, a recent large cross-sectional analysis of 7 adult cohorts from Europe and Africa observed that plasma concentrations of DPA and DHA were positively associated with FEV_1_ and FVC (45), with stronger associations in males and in smokers. The overall estimates were, however, rather small (e.g., 18.6-mL higher FEV_1_ per SD increase in DHA), with some variations across studies.

### Potential mechanisms

n–3 PUFAs may protect against asthma and other allergic diseases through their anti-inflammatory properties. n–3 PUFAs can lower inflammation through several different pathways including inhibition of eicosanoids (e.g., prostaglandins and leukotrienes) and through production of inflammation-resolving resolvins (46). It has also been suggested that allergic individuals may have an altered fatty acid metabolism, with lower blood concentrations of VLC PUFAs, despite similar intake (47, 48). However, in the BAMSE study, we have previously compared the correlation between dietary intake and plasma concentrations of PUFAs between allergic and nonallergic individuals and found no differences (11). In addition, the longitudinal analyses of incident asthma further indicate that PUFA concentrations influence the risk of asthma and not the opposite.

In contrast to n–3 PUFAs, n–6 PUFAs were previously often described as proinflammatory, although little evidence exists to support such effects in humans, because several randomized feeding studies have shown that even overfeeding with dietary LA or intake at high doses does not cause any increase in inflammatory markers, despite a several-fold increase in the dietary n–6:n–3 ratio (49, 50). Notably, the role of n–6 PUFAs in inflammation has been shown to be complex, with both proinflammatory and anti-inflammatory properties, and dependent on the type of n–6 fatty acid (8, 33).

### Strengths and limitations

The strength of the present study is the population-based prospective cohort design with repeated measurements of both plasma phospholipid concentrations of fatty acids and self-reported dietary intake. In addition, the long follow-up period enabled longitudinal analyses from childhood up to adulthood. We were further able to take into account potential disease-related modification of exposure by adjusting for early symptoms of allergic disease as well as to exclude participants who avoided fish because of allergy. Some potential limitations should be highlighted. First, although plasma fatty acids were only available in a subset of our participants, selection bias is not likely to have influenced the observed results, because the distribution of background factors was similar to that among the full cohort. Second, both asthma and dietary intakes were self-reported, although any misclassification is probably nondifferential and may only have resulted in attenuation of the associations (51). Finally, although we evaluated the impact of several potential confounders, we cannot exclude residual or unmeasured confounding.

### Conclusion

In conclusion, this longitudinal study of a population-based cohort shows that, in childhood and adolescence, higher plasma proportions of the n–3 PUFA ALA and VLC n–3 PUFAs, as well as the 2 n–6 PUFAs LA and AA, were associated with reduced risk of asthma up to young adulthood. Because these PUFAs measured in plasma overall are established biomarkers of dietary intake, our results may suggest that higher intakes of these PUFAs are associated with lowered asthma risk. In contrast, higher self-reported dietary intakes of n–6 PUFAs were associated with increased risk of asthma at 24 y. No consistent associations were observed with lung function. Future studies should focus on increasing the understanding regarding potential mechanisms behind these associations, which may contribute to strengthening dietary guidelines and to interventions to prevent asthma in the general population.

## Supplementary Material

nqab427_Supplemental_File

## Data Availability

Data described in the article, codebook, and analytic code will be made available upon request in case of an approved application and establishment of relevant agreements.

## Notes

Supported by grants from the Swedish Research Council; the Swedish Research Council for Health, Working Life and Welfare; Formas; the Asthma and Allergy Association; the Swedish Heart-Lung Foundation; the European Research Council (TRIBAL, grant agreement 757919, to EM); the Swedish Asthma and Allergy Research Foundation; and Region Stockholm (ALF project, and for cohort and database maintenance). Thermo Fisher Scientific kindly provided reagents for IgE analyses. The funding sources had no involvement or restrictions regarding publication.

Supplemental Methods, Supplemental Figure 1, and Supplemental Tables 1–6 are available from the “Supplementary data” link in the online posting of the article and from the same link in the online table of contents at https://academic.oup.com/ajcn/.

Abbreviations used: AA, arachidonic acid; ALA, α-linolenic acid; BAMSE, Barn, Allergi, Miljö, Stockholm, Epidemiologi; DPA, docosapentaenoic acid; *FADS*, fatty acid desaturase; FEV_1_, forced expiratory volume in 1 s; FVC, forced vital capacity; LA, linoleic acid; VLC, very-long-chain.
