# Supplementary material for: Dietary intake and plasma concentrations of PUFAs in childhood and adolescence in relation to asthma and lung function up to adulthood
Source: Am J Clin Nutr. 2021 Dec 29;115(3):886–96. doi: 10.1093/ajcn/nqab427 (PMC8895221; doi:10.1093/ajcn/nqab427)
Supplement: nqab427_Supplemental_File [file nqab427_supplemental_file.docx]

**Online Supplementary Material**

# **Dietary and plasma levels of polyunsaturated fatty acids in childhood and adolescence in relation to asthma and lung function up to adulthood**

Sandra Ekström^1,2^, Emmanouela Sdona^2^, Susanna Klevebro^3,4^, Jenny Hallberg^2,4^, Antonios Georgelis^1,2^, Inger Kull^3,4,^, Erik Melén^2,3,4^, Ulf Risérus^5^, Anna Bergström^1,2^

^1^ Center for Occupational and Environmental Medicine, Region Stockholm, Stockholm, Sweden

^2^ Institute of Environmental medicine, Karolinska Institutet, Stockholm, Sweden

^3^ Department of Clinical Science and Education, Södersjukhuset, Karolinska Institutet, Stockholm, Sweden

^4^ Sachs' Children and Youth Hospital, Södersjukhuset, Stockholm, Sweden

^5^ Department of Public Health and Caring Sciences, Clinical Nutrition and Metabolism, Uppsala University, Uppsala, Sweden

## **Supplementary Methods**

## **Analyses of fatty acids in plasma phospholipids**

Fatty acids in plasma phospholipids were analyzed at 8 years (n=940) and 16 years (n=939) using gas chromatography as previously described (1). Levels were expressed in relative amounts as proportion of total fatty acids of the 15 different fatty acids obtained: myristic acid (14:0), Pentadecyclic acid (15:0), Palmitic acid (16:0), Palmitoleic acid (16:1), Margaric acid (17:0), Stearic acid (18:0), Oleic acid (18:1), Linoleic acid (LA, 18:2), γ-Linolenic acid (18:3), α-linolenic acid (ALA; 18:3n-3), Dihomo-γ-linoleic acid (20:3), Arachidonic acid (AA, 20:4), Eicosapentaeonic acid (EPA, 20:5), Docosapentaeonic acid (DPA, 22:5), Docosahexaeonic acid (DHA, 22:6).

## **Lung function measurements**

## Lung function was measured by spirometry in 2,613 participants at 8 years using a 2200 Pulmonary Function Laboratory (Sensormedics, Anaheim, CA) spirometer, in 2,312 participants at 16 years using a Jaeger MasterScreen-IOS system (Carefusion Technologies, San Diego, CA) and in 2,212 participants at 24 years using a Vyaire Vyntus system (Vyaire Medical, IL, USA). Participants performed repeated maximal expiratory flow volume (MEFV) measurements. The curves were manually inspected and evaluated according to the American Thoracic Society (ATS) and European Respiratory Society (ERS) criteria (2).

## **Definition of covariates**

Living area at birth: Urban areas included central parts of Stockholm (Norrmalm). Suburban areas included northwestern parts of Stockholm county (the municipalities Järfälla, Solna and Sundbyberg).

Allergic heredity: Doctor diagnosed asthma and/or hay fever in combination with reported allergy to pollen or pets in one or both parents (assessed in the baseline questionnaire).

Parental occupation: Parental occupation was categorized as professional worker (white collar worker) or non-professional worker (blue collar worker) according to Statistics Sweden “Socioeconomic division (SEI); Reports on Statistical Coordination 1982:4” (assessed in the baseline questionnaire).

Maternal smoking during pregnancy and/or infancy: The mother smoked at least one cigarette per day at any point of time during pregnancy and/or in infancy (assessed in the baseline questionnaire).

Parental origin outside of Scandinavia: one or both parents born outside of Sweden, Norway, Denmark, or Finland (assessed in the 8-year questionnaire).

Breastfeeding ≥ 4 months: Exclusive breastfeeding for at least four months (assessed in the 1-year questionnaire).

Maternal BMI in early pregnancy: Maternal body mass index (kg/m^2^) recorded at the first visit to the antenatal-care clinic around week 10 in pregnancy (obtained from the Swedish Medical Birth Register).

Fish intake at 1y: Frequency of total fish intake, categorized as ≥2 times /month and <2 times/month (assessed in the 1-year questionnaire).

Omega-3 supplements: regular or irregular use of omega-3 supplements (assessed in the 16-year questionnaire).

Smoking: Daily or occasional smoking (assessed in the 16-year and 24-year questionnaire).

Physical activity: Self-reported amount of vigorous (e.g. lifting heavy weights, aerobics, or high-speed bicycling) and moderate (e.g. bicycling at normal speed, carrying light objects) physical activity (hours/week) in the last 12 months. Mean of summer and winter season was calculated. Levels of physical activity was defined according to IPAQ guidelines(3). High levels of physical activity: ≥7hours/week of moderate to vigorous activity or ≥ 3.5 hours/week of vigorous activity (obtained from the 16-year and 24-year questionnaire).

Overweight: Weight and height was measured at clinical investigations at 8, 16 and 24 years. At 8 years and 16 years, overweight was defined per gender- and age-specific cut-off values for body mass index proposed by the International Obesity Task Force (4). At 24 years, overweight was defined as body mass index ≥ 25 kg/m^2^.

FADS genotype: Fatty acid desaturase (*FADS*) single nucleotide polymorphism (SNP, rs1535) was analyzed through GWAS performed at 8 and 16 years blood sample using Illumina 610k and Ilumina Infinium Global Screening Array-24.10 BeadChip following the same sample collection procedures, protocols and questionnaires. The details of the analyses have been described elsewhere (5). In the present study, the minor G allele homozygous was analyzed together with the major A allele heterozygotes, due to few individuals (GG and GA vs. AA).


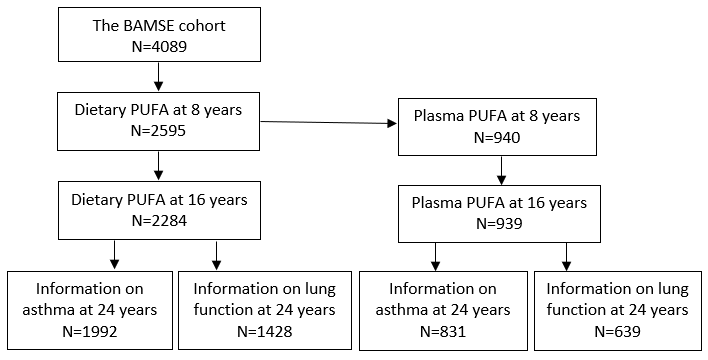


##

## **Supplementary Figure 1.** Overview of the included participants in the study populations.

## PUFA: polyunsaturated fatty acids.

| **Supplementary Table 1.** Description of the study populations included in the analyses on dietary and plasma levels of PUFA and asthma compared to the total cohort. | | | |
| --- | --- | --- | --- |
|  | Information on dietary PUFA at 8, 16 y and asthma at 24 y (n=1992) | Information on  plasma PUFA at 8, 16 y and asthma 24 y (n=831) | **Total cohort**  (n=4089) |
| **Variable^1^** | **n (%)** | **n (%)** | **n (%)** |
| **Male sex** | 952 (47.8) | 363 (44.0) | 2065 (50.5) |
| **Living area at birth^2^**   - Urban - Suburban | 656 (33.1)  1327 (66.9) | 270 (32.7)  557 (67.4) | 1205 (29.6)  2866 (70.4) |
| **Parental professional worker** | 1702 (86.4) | 728 (88.1) | 3323 (82.7) |
| **Maternal overweight in early pregnancy** | 349 (20.3) | 149 (20.8) | 724 (20.4) |
| **Maternal smoking in pregnancy and/or infancy** | 240 (12.1) | 94 (11.3) | 563 (13.8) |
| **Parental origin outside Scandinavia** | 310 (15.7) | 133 (16.0) | 543 (16.0) |
| **Breastfeeding ≥4 months** | 1591 (81.3) | 656 (80.4) | 3116 (79.5) |
| **Fish intake in infancy ≥ 2 times/month** | 1576 (80.5) | 659 (80.8) | 3143 (80.1) |

PUFA: polyunsaturated fatty acid

1. Numbers may not add up to total due to internal missing
2. Urban: central parts of Stockholm (Norrmalm); suburban: northwestern parts of Stockholm county (the municipalities Järfälla, Solna or Sundbyberg)

PUFA: polyunsaturated fatty acid

| **Supplementary Table 2**. Description of dietary and plasma PUFA at 8 and 16 years, by sex, among participants in the study populations (n=1992 for dietary PUFA and n=831 for plasma PUFA) | | | | | | |  |
| --- | --- | --- | --- | --- | --- | --- | --- |
|  | **Females** | | **Males** | | **Total** | |  |
| **Dietary PUFAs at 8 years (g/1900 kcal)** | **median** | **25-75^th^ perc.** | **median** | **25-75^th^ perc.** | **median** | **25-75^th^ perc.** | **p-value^1^** |
| ALA | 1.15 | 1.00-1.34 | 1.14 | 1.00-1.33 | 1.14 | 1.00-1.33 | 0.29 |
| ∑VLC n-3 | 0.24 | 0.14-0.32 | 0.22 | 0.13-0.30 | 0.24 | 0.14-0.31 | 0.02 |
| LA | 6.30 | 5.60-7.16 | 6.20 | 5.78-7.02 | 6.25 | 5.60-7.09 | 0.08 |
| AA | 0.07 | 0.06-0.09 | 0.07 | 0.06-0.09 | 0.07 | 0.06-0.09 | 0.15 |
| n-6/n-3 ratio | 4.55 | 4.17-4.94 | 4.58 | 4.18-4.97 | 4.57 | 4.17-4.96 | 0.69 |
| **Dietary PUFAs at 16 years (g/1900 kcal)** |  |  |  |  |  |  |  |
| ALA | 1.32 | 1.13-1.53 | 1.33 | 1.15-1.52 | 1.33 | 1.14-1.53 | 0.67 |
| ∑VLC n-3 | 0.27 | 0.14-0.40 | 0.27 | 0.16-0.41 | 0.27 | 0.15-0.40 | 0.07 |
| LA | 8.02 | 6.66-9.75 | 7.63 | 6.62-9.06 | 7.85 | 6.64-9.47 | <0.001 |
| AA | 0.07 | 0.05-0.10 | 0.08 | 0.06-0.11 | 0.08 | 0.06-0.11 | <0.001 |
| n-6/n-3 ratio | 4.99 | 4.29-5.90 | 4.71 | 4.13-5.55 | 4.84 | 4.22-5.75 | <0.001 |
| **Plasma proportions of PUFA at 8 years (% of total fatty acids)** |  |  |  |  |  |  |  |
| ALA | 0.23 | 0.19-0.28 | 0.24 | 0.19-0.29 | 0.23 | 0.19-0.28 | 0.35 |
| ∑VLC n-3 | 3.30 | 2.59-3.95 | 3.33 | 2.67-4.13 | 3.32 | 2.60-4.06 | 0.39 |
| LA | 21.31 | 20.09-22.58 | 21.11 | 19.79-22.66 | 21.23 | 20.00-22.59 | 0.17 |
| AA | 5.51 | 4.54-6.44 | 5.78 | 4.70-6.71 | 5.61 | 4.65-6.60 | 0.02 |
| **Plasma proportion of PUFA at 16 years (% of total fatty acids)** |  |  |  |  |  |  |  |
| ALA | 0.29 | 0.24-0.34 | 0.27 | 0.23-0.33 | 0.28 | 0.23-0.34 | 0.006 |
| ∑VLC n-3 | 5.46 | 4.61-6.42 | 5.29 | 4.75-6.06 | 5.38 | 4.65-6.25 | 0.29 |
| LA | 21.82 | 20.62-23.23 | 21.88 | 20.49-23.25 | 21.85 | 20.51-23.24 | 0.88 |
| AA | 8.67 | 7.78-9.66 | 9.12 | 8.32-10.07 | 8.89 | 8.04-9.88 | <0.001 |
| 1. P-value comparing females vs. males was obtained by the Wilcoxon rank-sum test   PUFA: polyunsaturated fatty acid  ALA: a-linolenic acid (18:3 n-3), ∑VLC n-3: sum of very long chain n-3 fatty acids (EPA: eicosapentaenoic acid (20:5 n-3), DPA: docosapentaenoic acid (22:5 n-3), DHA: docosahexaenoic acid (22:6 n-3), LA: linoleic acid (18:2 n-6), AA: arachidonic acid (20:4 n-6), n-6/n-3 ratio: sum of LA and AA divided by sum of ALA, EPA, DPA and DHA. | | | | | | |  |

| **Supplementary Table 3.** Correlation between plasma fatty acids at 16 years in the study population (n=831) | | | | | | |
| --- | --- | --- | --- | --- | --- | --- |
|  | **∑VLC n-3** | | **ALA** | | **LA** | |
|  | **r^1^** | **p-value^2^** | **r^1^** | **p-value^2^** | **r^1^** | **p-value^2^** |
| ALA | -0.06 | 0.61 |  |  |  |  |
| LA | -0.35 | <0.001 | 0.28 | <0.001 |  |  |
| AA | 0.29 | <0.001 | -0.33 | <0.001 | -0.35 | <0.001 |
| ^1^ Spearman correlation coefficient  ^2^ p-values Bonferroni corrected  ALA: a-linolenic acid (18:3 n-3), ∑VLC n-3: sum of very long chain n-3 fatty acids (EPA: eicosapentaenoic acid (20:5 n-3), DPA: docosapentaenoic acid (22:5 n-3), DHA: docosahexaenoic acid (22:6 n-3), LA: linoleic acid (18:2 n-6), AA: arachidonic acid (20:4 n-6) | | | | | | |

| **Supplementary Table 4.** Correlation between dietary and plasma PUFA at 16 years in the study population (n=812) | | |
| --- | --- | --- |
|  | **16 years** | |
|  | **r^1^** | **p-value** |
| **n-3 FA** |  |  |
| ALA | -0.04 | 0.21 |
| EPA | 0.25 | <0.001 |
| DPA | -0.01 | 0.75 |
| DHA | 0.31 | <0.001 |
| ∑VLC n-3 | 0.31 | <0.001 |
| **n-6 FA** |  |  |
| LA | 0.06 | 0.08 |
| AA | 0.05 | 0.15 |

^1^ Spearman rank correlation between plasma fatty acid (% of total fatty acid) and energy adjusted dietary fatty acid

PUFA: polyunsaturated fatty acid

ALA: a-linolenic acid (18:3 n-3), ∑VLC n-3: sum of very long chain n-3 fatty acids (EPA: eicosapentaenoic acid (20:5 n-3), DPA: docosapentaenoic acid (22:5 n-3), DHA: docosahexaenoic acid (22:6 n-3), LA: linoleic acid (18:2 n-6), AA: arachidonic acid (20:4 n-6)

| **Supplementary Table 5.** Description of mean plasma proportions of very long-chain omega-3 (VLC n-3)^1^ polyunsaturated fatty acids (PUFA) at 8 and 16 years in relation to baseline and lifestyle characteristics (n=831). | | |
| --- | --- | --- |
|  | **Mean plasma level of ∑VLC n-3 PUFAs at 8 and 16 years (%)** |  |
|  | **Median (25^th^ – 75^th^)** | **P-value^2^** |
| **Living area at birth**   - Urban (n=270)^3^ - Suburban (n=557)^3^ | 4.66 (4.02-5.34)  4.30 (3.70-4.94) | <0.001 |
| **Parental professional worker at baseline**   - No (n=98) - Yes (n=728) | 4.09 (3.65-4.73)  4.46 (3.82-5.16) | 0.005 |
| **Maternal smoking in pregnancy and/or infancy**   - No (n=736) - Yes (n=94) | 4.44 (3.81-5.15)  4.22 (3.55-4.92) | 0.01 |
| ***FADS* genotype**   - AA (n=280) - GA/GG (n=482) | 4.58 (4.02-5.27)  4.30 (3.69-4.92) | <0.001 |
| **Fish intake at 8 years**   - <2 times/week (n=498) - ≥2 times/week (n=333) | 4.33 (3.73-5.02)  4.48 (3.85-5.27) | 0.009 |
| **Fish intake at 16 years**   - < 1-2 times/week (n=251) - ≥1-2 times/week (n=579) | 4.07 (3.48-4.65)  4.53 (3.93-5.30) | <0.001 |
| **Overweight at 24 years**   - No (n=522) - Yes (n=164) | 4.45 (3.84-5.12)  4.28 (3.68-4.87) | 0.04 |

1. ∑VLC n-3: sum of very long chain n-3 fatty acids (EPA: eicosapentaenoic acid (20:5 n-3), DPA: docosapentaenoic acid (22:5 n-3), DHA: docosahexaenoic acid (22:6 n-3)
2. P-value was obtained by the Wilcoxon rank-sum test
3. Urban: central parts of Stockholm (Norrmalm); suburban: northwestern parts of Stockholm county (the municipalities Järfälla, Solna or Sundbyberg).
4. Doctor diagnosed asthma and/or hay fever in combination with reported allergy to pollen or pets in one or both parents (assessed in the baseline questionnaire).

PUFA: polyunsaturated fatty acids.

| **Supplementary Table 6.** Descriptive results on asthma and lung function in the study population | | | | | | |
| --- | --- | --- | --- | --- | --- | --- |
| **Study population with dietary PUFA (n=1992 for asthma and n=1428 for lung function)** | | | | | | |
|  | **Females** | | **Males** | | **Total** | |
| **Asthma** | **n** | **%** | **n** | **%** | **n** | **%** |
| Prevalent asthma 8 years | 94/1036 | 9.1 | 116/944 | 12.3 | 210/1980 | 10.6 |
| Prevalent asthma 16 years | 165/1032 | 16.0 | 141/946 | 14.9 | 306/1978 | 15.5 |
| Prevalent asthma 24 years | 166/1040 | 16.0 | 125/952 | 13.1 | 291/1992 | 14.6 |
| Incident asthma 8-24 years | 103/942 | 10.9 | 66/828 | 8.0 | 169/1770 | 9.6 |
| **Lung function 24 years** | **n** | **Mean (SD)** | **n** | **Mean (SD)** |  |  |
| FEV_1_ (ml) | 794 | 3506 (430) | 634 | 4743 (629) |  |  |
| FVC (ml) | 794 | 4145 (526) | 634 | 5850 (810) |  |  |
| FEV_1_/FVC (%) | 794 | 84.8 (5.8) | 634 | 81.4 (6.1) |  |  |
|  |  |  |  |  |  |  |
| FEV_1_ (z-scores) | 794 | -0.16 (0.84) | 634 | -0.34 (0.89) |  |  |
| FVC (z-scores) | 794 | 0.01 (0.84) | 634 | -0.08 (0.88) |  |  |
| FEV_1_/FVC (z-scores) | 794 | -0.30 (0.89) | 634 | -0.44 (0.90) |  |  |
| **Study population with plasma PUFA (n=831 for asthma and n=639 for lung function)** | | | | | | |
| **Asthma** | **n** | **%** | **n** | **%** | **n** | **%** |
| Prevalent asthma 8 years | 54/463 | 11.7 | 57/363 | 15.7 | 111/826 | 13.4 |
| Prevalent asthma 16 years | 86/464 | 18.5 | 61/366 | 16.7 | 147/830 | 16.9 |
| Prevalent asthma 24 years | 89/465 | 19.1 | 53/366 | 14.5 | 142/831 | 17.1 |
| Incident asthma 8-24 years | 50/409 | 12.2 | 26/306 | 8.5 | 76/715 | 10.6 |
| **Lung function 24 years** | **n** | **Mean (SD)** | **n** | **Mean (SD)** |  |  |
| FEV_1_ (ml) | 389 | 3507 (427) | 250 | 4720 (598) |  |  |
| FVC (ml) | 389 | 4164 (522) | 250 | 5870 (784) |  |  |
| FEV_1_/FVC (%) | 389 | 84.5 (5.9) | 250 | 80.7 (6.0) |  |  |
|  |  |  |  |  |  |  |
| FEV_1_ (z-scores) | 389 | -0.17 (0.84) | 250 | -0.39 (0.87) |  |  |
| FVC (z-scores) | 389 | 0.04 (0.85) | 250 | -0.06 (0.85) |  |  |
| FEV_1_/FVC (z-scores) | 389 | -0.35 (0.88) | 250 | -0.54 (0.86) |  |  |

PUFA: polyunsaturated fatty acids, FEV_1_ Forced expiratory volume in 1 second, FVC: Forced vital capacity

**References**

1. Magnusson J, Ekström S, Kull I, Håkansson N, Nilsson S, Wickman M, Melén E, Risérus U, Bergström A. Polyunsaturated fatty acids in plasma at 8 years and subsequent allergic disease. J Allergy Clin Immunol 2018;142(2):510-6.e6. doi: 10.1016/j.jaci.2017.09.023.

2. Miller MR, Hankinson J, Brusasco V, Burgos F, Casaburi R, Coates A, Crapo R, Enright P, van der Grinten CP, Gustafsson P, et al. Standardisation of spirometry. Eur Respir J 2005;26(2):319-38. doi: 10.1183/09031936.05.00034805.

3. Guidelines for data processing analysis of the International Physical Activity Questionnaire (IPAQ) - Short and long forms. 2005 2015-01-29 <http://www.ipaq.ki.se/scoring.pdf>.

4. Cole TJ, Lobstein T. Extended international (IOTF) body mass index cut-offs for thinness, overweight and obesity. Pediatr Obes 2012;7(4):284-94. doi: 10.1111/j.2047-6310.2012.00064.x.

5. Talaei M, Sdona E, Calder PC, Jones LR, Emmett PM, Granell R, Bergström A, Melén E, Shaheen SO. Intake of n-3 polyunsaturated fatty acids in childhood, *FADS* genotype, and incident asthma. European Respiratory Journal 2021:2003633. doi: 10.1183/13993003.03633-2020.
